# Simvastatin Therapy for Drug Repositioning to Reduce the Risk of Prostate Cancer Mortality in Patients With Hyperlipidemia

**DOI:** 10.3389/fphar.2018.00225

**Published:** 2018-03-22

**Authors:** Yu-An Chen, Ying-Ju Lin, Cheng-Li Lin, Hwai-Jeng Lin, Hua-Shan Wu, Hui-Ying Hsu, Yu-Chen Sun, Hui-Yu Wu, Chih-Ho Lai, Chia-Hung Kao

**Affiliations:** ^1^Graduate Institute of Basic Medical Science, School of Medicine, College of Medicine, China Medical University, Taichung, Taiwan; ^2^Department of Medical Research, School of Chinese Medicine, China Medical University and Hospital, Taichung, Taiwan; ^3^Management Office for Health Data, China Medical University Hospital, Taichung, Taiwan; ^4^Division of Gastroenterology and Hepatology, Department of Internal Medicine, School of Medicine, College of Medicine, Taipei Medical University, Taipei, Taiwan; ^5^Division of Gastroenterology and Hepatology, Department of Internal Medicine, Shuang-Ho Hospital, New Taipei City, Taiwan; ^6^Department of Nursing, Asia University, Taichung, Taiwan; ^7^Department of Laboratory Medicine, Chang Gung Memorial Hospital, Linkou, Taiwan; ^8^Department of Microbiology and Immunology, Graduate Institute of Biomedical Sciences, College of Medicine, Chang Gung University, Taoyuan, Taiwan; ^9^Molecular Infectious Disease Research Center, Chang Gung Memorial Hospital, Linkou, Taiwan; ^10^Department of Bioinformatics and Medical Engineering, Asia University, Taichung, Taiwan; ^11^Department of Nuclear Medicine, PET Center, China Medical University Hospital, Taichung, Taiwan

**Keywords:** hyperlipidemia, HMG-CoA reductase, prostate cancer, statin, cohort study

## Abstract

Prostate cancer (PCa) is one of the most commonly diagnosed cancers in the western world, and the mortality rate from PCa in Asia has been increasing recently. Statins are potent inhibitors of 3-hydroxy-3-methyl glutaryl coenzyme A (HMG-CoA) reductase and are commonly used for treating hyperlipidemia, with beneficial effects for cardiovascular disease and they also exhibit anti-cancer activity. However, the protective effects of statins against PCa are controversial. In this study, we investigated the effect of two types of statins (simvastatin and lovastatin) and the mortality rate of PCa patients by using the Taiwan National Health Insurance Research Database (NHIRD). A total of 15,264 PCa patients with hyperlipidemia records and medical claims from the Registry of Catastrophic Illness were enrolled. The patients were divided into two cohorts based on their statin use before the diagnosis of PCa: statin users (*n* = 1,827) and non-statin users (*n* = 1,826). The results showed that patients who used statins exhibited a significantly reduced risk of mortality from PCa [adjusted hazard ratio (HR) = 0.84, 95% CI = 0.73–0.97]. Analysis of the cumulative defined daily dose (DDD) indicated that patients who were prescribed simvastatin ≥ 180 DDD had a dramatically decreased risk of death from PCa (adjusted HR = 0.63; 95% CI = 0.51–0.77). This population-based cohort study demonstrated that statin use significantly decreased the mortality of PCa patients, and that this risk was inversely associated with the cumulative DDD of simvastatin therapy. The results of this study revealed that statins may be used for drug repositioning and in the development of a feasible approach to prevent death from PCa.

## Introduction

Prostate cancer (PCa) is one of the most prevalent male cancers in western developed countries, and its incidence is increasing in the Asia-Pacific region (Global Burden of Disease Cancer et al., 2017). Although recent advances in surgical techniques and radiotherapy have improved the disease-free survival rates of men with localized PCa, the recurrence rate after radical prostatectomy, chemotherapy, or radiotherapy is still high (Trock et al., 2008). Men are at higher risk for developing PCa, while the risk factors include having a family history of PCa along with environmental and lifestyle-related factors, such as physical inactivity, obesity, and a high-fat diet (Barnard, 2007; Meyerhardt et al., 2010).

Statins, inhibitors of 3-hydroxy-3-methyl glutaryl coenzyme A (HMG-CoA) reductase, the rate-limiting enzyme in cholesterol biosynthesis, are widely used for treating high levels of serum cholesterol (Hebert et al., 1997). The activity of HMG-CoA reductase may be increased due to the activation of transcriptional regulation mediated by sterol regulatory element binding proteins (SREBPs) (Chen et al., 1978). In LNCaP and PC-3 PCa cells, inhibition of SREBP2 activity reduced cell viability (Krycer et al., 2012). Recent studies indicated that statins possess the potential to prevent and treat several types of cancers, including PCa (Hindler et al., 2006; Bonovas et al., 2008; Yu et al., 2014). The protective functions of statins are included reducing the risk of PCa through arrest cell cycle, inducing apoptosis, and inhibiting tumor metastasis (Papadopoulos et al., 2011; Sun Q. et al., 2015). However, some conflicting results about the protective effects of statins against PCa have not been clearly clarified (Moon et al., 2014; Alfaqih et al., 2017).

Although previous studies indicated that statins may decrease the incidence of PCa (Shannon et al., 2005; Flick et al., 2007), there have been no large-scale epidemiologic studies on the effects of statins for treating PCa patients with hyperlipidemia who received different therapeutic modalities including radical prostatectomy, chemotherapy, or radiotherapy. In this study, we conducted a nationwide population-based cohort study to analyze the mortality in PCa patients with hyperlipidemia who were prescribed either simvastatin or lovastatin. The association between the cumulative defined daily dose (DDD) of two types of statins and the risk of PCa mortality was also investigated. Our results showed that statin prescriptions effectively reduced the risk of death from PCa. This study also revealed that cholesterol-lowering agents, such as statins, may be used for drug repurposing to help prevent death in PCa patients who also have hyperlipidemia.

## Materials and Methods

### Data Source

To examine whether statin use is associated with risk of PCa, a nationwide cohort study was conducted. Data were obtained from the Registry of Catastrophic Illness and Taiwan National Health Insurance Research Database (NHIRD). The National Health Research Institutes (NHRI) in Taiwan is responsible for managing the insurance claims data reported to the Bureau of Health Insurance. For research purposes, the NHRI compiles all medical claims in the National Health Insurance (NHI) program and releases the database annually to the public. Patient consent is not required to access the NHIRD. This study was approved by the Institutional Review Board of China Medical University in Taiwan (CMUH104-REC2-115-CR2).

### Population-Based Cohort Study

**FIGURE 1:**
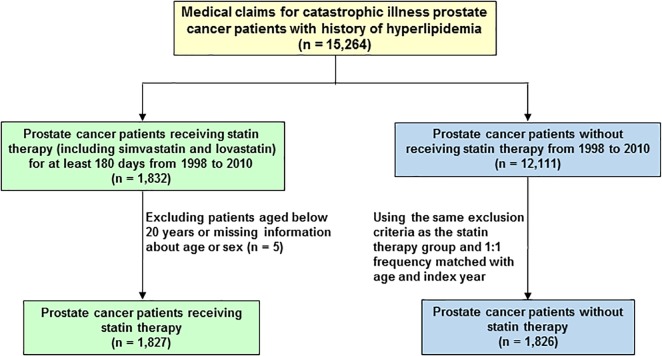
Flowchart of the establishment of cohorts, patient selection, identification, and analysis.

A total of 15,264 PCa patients with hyperlipidemia (ICD-9-CM 272) were included in this study (**Figure 1**). The patients were divided into two cohorts, the statin users (either simvastatin or lovastatin) and non-statin users before the diagnosis of PCa. Patients under 20 years old were excluded. In the statin use cohort, 1,827 PCa patients prescribed statins for at least 6 months during January 1, 1998 to December 31, 2010. The non-statin cohort (*n* = 1,826) was randomly selected from 12,111 PCa patients without receiving statin therapy. Comparison group was established by 1:1 randomly frequency matching (according to age and index year) with PCa patients who did not use any types of statin-based drugs during the study period. The study endpoint was mortality.

### Medication and Analysis of Statin Prescription

Hazard ratio (HR) and 95% confidence interval (CI) were adjusted for age, treatment of hormone therapy (including oral and injection), radical prostatectomy, radiotherapy, chemotherapy, and the comorbidities of diabetes (ICD-9-CM codes 250), hypertension (ICD-9-CM codes 401- 405), stroke (ICD-9-CM codes 430-438), cardiovascular disease (CAD; ICD-9-CM codes 410–414) and chronic obstructive pulmonary disease (COPD; ICD-9-CM codes 490–496).

The DDD, recommended by the World Health Organization (WHO), was assumed to be the average maintenance dose per day of a drug and analyzed as described previously (Lin et al., 2017). The cumulative DDD was calculated by deriving the total prescribed DDD of each type of statin, namely simvastatin (ATC C10AA01) and lovastatin (ATC C10AA02), for statin users. For each statin type, the cumulative DDD was partitioned into two levels by setting the cutoff value in the median.

### Statistical Analysis

The distributions of demographic characteristics were compared between the statin and non-statin cohorts, and the differences were examined using the Chi-squared test for categorical variables and the Student’s *t*-test for continuous variables. The multivariate analysis of the mortality rates associated with statin use was performed using the cox proportional hazard regressions. Furthermore, we divided each statin type into two groups according to the quartile of cumulative DDD. Cox proportional hazard regression was used to assess how the statin dose affected the relative risk of mortality. A *P*-value of < 0.05 indicated statistical significance. All analyses were conducted using SAS statistical software (Version 9.3 for Windows; SAS Institute, Inc., Cary, NC, United States).

## Results

### Demographic Characteristics of Study Patients

A total of 15,264 PCa patients with hyperlipidemia were enrolled in this study and analyzed. In the population-based cohort study, we first evaluated 3,653 patients with PCa, aged ≥ 20 years (**Figure 1**). There were 1,827 patients using statin-based drugs (either simvastatin or lovastatin) and 1,826 patients who did not. **Table 1** shows the age, comorbidities, hormone therapy, and treatments of the cohorts. Among the patients with PCa, 85.4% of the patients were older than 60 years of age. Compared with non-statin users (Group 1), the statin users (Group 2) were more likely to have coronary artery disease (CAD) (41.2% vs. 52.2%, *P* < 0.0001), diabetes mellitus (20.2% vs. 32.3%, *P* < 0.0001), hypertension (68.9% vs. 81.3%, *P* < 0.0001), and stroke (5.37% vs. 7.88%, *P* < 0.0001). In the treatments used for PCa including hormone therapy, radical prostatectomy, chemotherapy, and radiotherapy, the statin users and non-statin users did not show statistically significant differences.

**Table 1:** Demographic characteristics of PCa patients with hyperlipidemia by medications.

Parameter	Group 1^a^	Group 2	*P*-value^b^
**Age, years, *n* (%)**			0.99
≤59	266 (14.6%)	267 (14.6%)	
60–69	747 (40.9%)	747 (40.9%)	
70–79	712 (39.0%)	712 (39.0%)	
≥80	101 (5.5%)	101 (5.5%)	
Mean (SD)	68.3 (8.1%)	68.3 (7.8%)	0.68
**Comorbidity^c^, *n* (%)**			
CAD	753 (41.2%)	954 (52.2%)	<0.0001
Diabetes mellitus	368 (20.2%)	590 (32.3%)	<0.0001
Hypertension	1258 (68.9%)	1486 (81.3%)	<0.0001
Stroke	98 (5.4%)	144 (7.9%)	<0.0001
COPD	675 (37.0%)	661 (36.2%)	0.62
**Hormone therapy, *n* (%)**			
Oral	780 (42.7%)	825 (45.2%)	0.14
Injection	215 (11.8%)	193 (10.6%)	0.25
**Treatment, *n* (%)**			
Radical prostatectomy	355 (19.4%)	379 (20.7%)	0.33
Chemotherapy	121 (6.6%)	100 (5.5%)	0.14
Radiotherapy	591 (32.4%)	609 (33.3%)	0.53


*^*a*^Group 1, non-statin users (*n* = 1826); group 2, statin users (*n* = 1827);*

*^*b*^Chi-square test and Student’s *t*-test were used to compare the statin user and non-statin user groups; ^*c*^CAD, coronary artery disease; COPD, chronic obstructive pulmonary disease.*

### Comparison of Incidence and Hazard Ratio (HR) of Mortality in PCa Patients

The person-years (PY), incidence (age, comorbidity, hormone therapy, and treatments), and HR for the risk of mortality among the cohorts were then analyzed. As shown in **Table 2**, statin users had a significantly lower HR of mortality (adjusted HR = 0.84, 95% CI = 0.73–0.97, *P* < 0.05) compared with the non-statin users. Among patients who had no comorbidities, statin users exhibited the lowest HR of mortality (adjusted HR = 0.55, 95% CI = 0.31–0.99, *P* < 0.05). A significantly reduced adjusted HR of mortality was also found in the following groups for patients prescribed statins: treatment with chemotherapy (adjusted HR = 0.60, 95% CI = 0.41–0.87, *P* < 0.01), non-oral hormone therapy (adjusted HR = 0.81, 95% CI = 0.68–0.98, *P* < 0.05), and non-prostatectomy (adjusted HR = 0.86, 95% CI = 0.74–0.99, *P* < 0.05).

**Table 2:** Hazard ratios and 95% confidence intervals of PCa mortality in statin user and non-statin user groups.

Variable	Group 1^a^			Group 2			Hazard ratio	
			
	Event	PY^b^	Rate	Event	PY	Rate	Crude	Adjusted HR^c^ (95% confidence interval)
**All**	402	14902	27.0	377	15119	24.9	0.91	0.84 (0.73–0.97)^∗^
**Age, years**								
≤59	22	2480	8.87	18	2516	7.15	0.80	0.83 (0.43–1.60)
60–69	123	6516	18.9	111	6584	16.9	0.88	0.90 (0.69–1.17)
70–79	217	5415	40.1	220	5508	40.0	0.98	0.88 (0.73–1.06)
≥80	40	492	81.3	28	512	54.7	0.63	0.67 (0.41–1.12)
**Comorbidity^d^**								
No	50	2494	20.0	16	1199	13.4	0.61	0.55 (0.31–0.99)^∗^
Yes	352	12408	28.4	316	13920	25.9	0.90	0.91 (0.79–1.05)
**Hormone therapy (Oral)**								
No	264	8437	31.3	227	8136	27.9	0.88	0.81 (0.68–0.98)^∗^
Yes	138	6465	21.3	150	6983	21.5	0.99	0.92 (0.72–1.16)
**Hormone therapy (Injection)**								
No	342	13156	26.0	331	13556	24.4	0.93	0.86 (0.74–1.01)
Yes	60	1747	34.4	46	1564	29.4	0.85	0.75 (0.50–1.12)
**Treatment**								
**Radical prostatectomy**								
No	363	11983	30.3	340	11871	28.6	0.94	0.86 (0.74–0.99)^∗^
Yes	39	2919	13.4	37	3249	11.4	0.82	0.67 (0.42–1.08)
**Chemotherapy**								
No	325	13942	23.3	328	14313	22.9	0.97	0.95 (0.81–1.11)
Yes	77	960	80.2	49	806	60.8	0.73	0.60 (0.41–0.87)^∗∗^
**Radiotherapy**								
No	260	9969	26.1	236	9971	23.7	0.90	0.84 (0.70–1.00)
Yes	142	4933	28.8	141	5148	27.4	0.93	0.88 (0.70–1.12)


*^*a*^Group 1, non-statin users; group 2, statin users; ^*b*^PY, per 1000 person-years; Rate, incidence rate; ^*c*^Adjusted HR, multivariable analysis including age, sex, hormone therapy, treatment and comorbidities of coronary artery disease (CAD), diabetes, hypertension, stroke, and chronic obstructive pulmonary disease (COPD); ^∗^*P* < 0.05; ^∗∗^*P* < 0.01; ^*d*^Comorbidity: only to have one of comorbidities classified as the comorbidity group.*

### Statin Use Reduces the Risk of PCa Mortality

The association between the cumulative DDD of two types of statins and the risk of PCa mortality was further analyzed. Compared with non-statin users, patients who were prescribed simvastatin at a DDD ≥ 180 (adjusted HR = 0.63, 95% CI = 0.51–0.77) exhibited the lowest risk of mortality, which was associated with the high cumulative DDD of prescribed simvastatin (**Table 3**). Patients who received lovastatin at a DDD < 115 (adjusted HR = 0.83, 95% CI = 0.71–0.97) had a lower adjusted HR of mortality compared with the non-statin user group. The results from these analyses demonstrate that statin use effectively reduced the mortality of PCa patients, and that this risk was associated with a high cumulative DDD of prescribed simvastatin.

**Table 3:** Prescribed statins reduce the mortality of PCa patients.

	Event/Total number	Crude HR^a^(95% confidence interval)	Adjusted HR^b^(95% confidence interval)
**Non-use of statins**	402/1826	1 (Reference)	1 (Reference)
**Use of statin^c^**			
**Simvastatin**			
<180 DDD	263/1101	1.11 (0.95–1.29)	0.99 (0.85–1.16)
≥180 DDD	114/726	0.65 (0.53–0.80)^∗∗∗^	0.63 (0.51–0.77)^∗∗∗^
**Lovastatin**			
< 115 DDD	259/1314	0.91 (0.78–1.06)	0.83 (0.71–0.97)^∗^
≥ 115 DDD	118/513	0.93 (0.76–1.14)	0.87 (0.71–1.07)


*^*a*^Crude HR, relative hazard ratio; ^*b*^Adjusted HR, multivariable analysis including age, sex, hormone therapy, treatment and comorbidities of coronary artery disease (CAD), diabetes, hypertension, stroke, and chronic obstructive pulmonary disease (COPD); ^*c*^The cumulative defined daily dose (DDD) is partitioned in to 2 segments by median; ^∗^*P* < 0.05; ^∗∗∗^*P* < 0.001.*

## Discussion

Prostate cancer is a commonly diagnosed cancer and the second leading cause of cancer-related deaths in US men (Wadajkar et al., 2013). In Taiwan, the prevalence of PCa has gradually increased in recent years (Chiang et al., 2016; Hung et al., 2016). Based on a statistical report from the Ministry of Health and Welfare [MOHW] (2016) of Taiwan in 2016, PCa has become the fifth most prevalent cancer and the seventh leading cause of cancer-related death, indicating that the threat of PCa to men has become severe. Therefore, it is important to pay more attention to the diagnosis and treatment of patients with PCa.

The regulation of lipid rafts (also referred to cholesterol-rich microdomains) is critical for PCa cell survival and growth (Zhuang et al., 2002). PCa cells harbored the activity to synthesize dihydrotestosterone (DHT) from acetic acid, indicating that the entire mevalonate-steroidogenic pathway was functionally intact (Locke et al., 2008). In addition, all enzymes contributed to testosterone and DHT synthesis are presented in many human primary and metastatic PCa (Montgomery et al., 2008). Therefore, new therapeutic agents with lesser toxicity and higher selectivity for preventing recurrence and progression of PCa are urgently required. This population-based cohort analysis revealed that statin therapy significantly decreased the mortality of PCa patients treated with chemotherapy. In addition, the reduced risk was correlated with the high cumulative DDD of prescribed simvastatin. This nationwide population-based study provides evidence that statin therapy not only reduced the risk of cardiovascular disease but may also be used for drug repurposing to prevent death of PCa patients who also have hyperlipidemia.

It has been demonstrated that simvastatin inhibits the proliferation, migration, and invasion of PCa cells via the up-regulation of ANXA10 and inactivation of the AKT/PI3K pathway (Miyazawa et al., 2017). Simvastatin can arrest the cell cycle at G1 in PC-3 cells through the AMPK pathway (Karlic et al., 2017). In addition, lovastatin was found to enhance the efficacy of PRRA-TRAIL via the promotion of tumor suppression and induction of apoptosis (Liu et al., 2015). A recent study also indicated that the induction of the LDL receptor is a possible mechanism of resistance that prostate tumors use to counteract the therapeutic effects of reducing serum cholesterol (Masko et al., 2017). Therefore, statins can be used to delay PCa progression by reducing LDL levels (Murtola et al., 2012).

Clinical studies have been reported that statins significantly reduced the incidence of advanced PCa (Bonovas et al., 2008). A 10-year retrospective cohort study indicated that statin users had a 31% lower risk of PCa (Farwell et al., 2011). The mechanism for statins was the indirect reduction in cellular cholesterol levels in multiple cell types through the lowering of circulating cholesterol, which impacts the membrane microdomains and steroidogenesis (Moon et al., 2014). In addition, statin can prevent against PCa by inhibiting the proliferation and inducing apoptosis of PCa cells, which inhibits angiogenesis, inflammation, and metastasis (Papadopoulos et al., 2011). A recent clinical analysis demonstrated that statin users had a significantly longer median time to progression during androgen deprivation therapy than non-users (Harshman et al., 2015).

Both *in vitro* and *in vivo* studies have demonstrated that statins inhibited cancer cell growth and induced apoptosis in a variety of tumor cell types, including PCa, colon adenocarcinoma, pancreatic carcinoma, and gastric cancer cells (Lochhead and Chan, 2013; Babcook et al., 2016; Lin et al., 2016; Gong et al., 2017). A population-based cohort study revealed that not all types of statin were associated with a decreased incidence of PCa, except for simvastatin, atorvastatin, and rosuvastatin (Lustman et al., 2014). Simvastatin was identified to inhibit the migration and invasion of PCa cells (Shah et al., 2016). The inhibitory effect of statin-derivatives on tumorigenicity of castrate-resistant prostate cancer (CRPC) may be mediated via concurrent inhibition of both androgen receptor and AKT signaling pathways, and membrane destabilization (Ingersoll et al., 2016). Furthermore, the mechanism for inhibition of CRPC growth by statins suppressed nuclear factor-κB pathway to induce apoptosis and prevent CRPC development (Park et al., 2013; Kang et al., 2017). Our current study also revealed that statins can function as a radiosensitizer in PCa cells for triggering the CHK1 checkpoint response and promoting DNA double-strand breaks (unpublish data). Together, these findings demonstrated the potential mechanism of statins in the treatment of PCa.

Although we recently reported the protective effects of statins against PCa (Sun L.M. et al., 2015), certain limitations and confounding factors of previous studies are still emerging. In the current study, we further selected patients with hyperlipidemia and divided them into two cohorts based on the prescription of statins. Because statin users may have other comorbidities, several treatments and comorbidities were adjusted for, including hormone therapy, diabetes, hypertension, stroke, CAD, and COPD. In addition, the cause of death among patients with PCa was documented in the Registry of Catastrophic Illness, and thus, mortality from PCa can be precisely analyzed. Therefore, our current findings have been thoroughly investigated and are based on rigorous and valid methods.

Some limitations exist in the current studies, including potential confounders, concomitant use of other chemo-preventive drugs, short enrollment periods, and small sample sizes. A social gradient in statin use may reflect social inequalities in health care use or health care quality, as well as systematic differences in factors such as smoking, nutrition, physical activity, and obesity (Bonovas and Sitaras, 2009). Patients with cardiovascular disease prescribed statins may also take non-steroidal anti-inflammatory drugs (NSAIDs), which have been reported to reduce the risk of PCa (Papadopoulos et al., 2011). Although current studies using population-based analysis consistently report an inverse association between statin use and PCa risk, the detailed mechanism of statins for the primary prevention of PCa requires further investigation.

The Taiwan government has launched an NHI program that has provided citizens with comprehensive coverage since 1995. All insurance claims are scrutinized by medical reimbursement specialists and recorded in the database. The NHIRD enabled appropriately selecting matched patients to represent the underlying population. In addition, we have analyzed data in the NHIRD to perform several studies and have evaluated statin-based drugs for protection against several diseases (Peng et al., 2015; Sun L.M. et al., 2015; Lin et al., 2016). Therefore, these results obtained by using a nationwide cohort study regarding statin use and diagnoses for patients with PCa mortality are reliable.

## Conclusion

This study demonstrated that statin prescription has effectively reduced the deaths of PCa patients with hyperlipidemia. The mortality risk of patients with PCa is inversely associated with a high cumulative DDD of simvastatin. Therefore, statins, cholesterol-lowering agents, can be drug repurposed and used in a preventive application for protecting against death from PCa. However, large, long-term clinical analysis and experimental studies on the biological mechanisms of statin therapy are required to further validate statins as a means of reducing PCa mortality.

## Author Contributions

C-HL and C-HK: conceived and designed the experiments. Y-AC, Y-JL, C-LL, H-JL, H-YH, Y-CS, and H-YW: performed the experiments and analyzed the data. Y-AC, Y-JL, C-LL, and H-JL: wrote the manuscript. All authors reviewed the final version of the manuscript.

## Conflict of Interest Statement

The authors declare that the research was conducted in the absence of any commercial or financial relationships that could be construed as a potential conflict of interest.
